# SORT as any easy technique to do; but needs more trials to be as a routine practice

**DOI:** 10.1186/s13613-020-00779-0

**Published:** 2020-12-02

**Authors:** Sarvin Sanaie, Ata Mahmoodpoor

**Affiliations:** 1 Neurosciences Research Center, Aging Research Institute, Tabriz University of Medical Sciences, Tabriz, Iran; 2 Department of Anesthesiology and Intensive Care, Faculty of Medicine, Tabriz University of Medical Sciences, Tabriz, Iran; 3 General ICU, Shohada Hospital, El-Goli Street, Tabriz, Iran

Najafi has published a letter about our published article regarding the comparison of SORT maneuver versus a conventional technique of neck flexion lateral pressure (NFLP) for nasogastric tube (NGT) insertion in ICU admitted patients [1]. He mentioned that there is a lack of standard definitions for the favorable outcome like the time span attributable to “ease of insertion” and the determinants for “insertion failure”. However, we tried to change the ease of insertion with our grading score which made it so easy to compare. We evaluated the ease of insertion with a 4-grade score as following: first grade as successful insertion in less than 50 s and in the first attempt, second grade as successful insertion in the first attempt with more than 50 s or in the second attempt with less than 100 s, third grade as successful insertion in the 2nd attempt with more than 100 s or in three attempts, and fourth grade as failed insertion. Regarding the rate of complications, based on the incidence of each complication, we need larger sample size to show the difference in each group but considering overall complications (35%), the mentioned sample size is sufficient to show the possible significant difference.

He also mentioned SORT maneuver as an ideal technique for NGT insertion in critically ill patients with COVID-19. As this technique considers anatomical characteristics, it can be an ideal option for health care workers who are familiar with this technique and airway anatomy. Moreover, every physician/nurses should notice the fact that NGT insertion is an aerosol producing technique and every one should consider full protection during its insertion with every technique [2]. So, SORT is an easy to learn technique; however, we should not forget the first rule in medicine: “Do not harm at first”.
